# Coenzyme A and protein CoAlation levels are regulated in response to oxidative stress and during morphogenesis in *Dictyostelium discoideum*

**DOI:** 10.1016/j.bbrc.2019.02.031

**Published:** 2019-04-02

**Authors:** Lujain Aloum, Christopher A. Brimson, Alexander Zhyvoloup, Robert Baines, Jovana Baković, Valeriy Filonenko, Christopher R.L. Thompson, Ivan Gout

**Affiliations:** a Department of Structural and Molecular Biology, University College London, London, WC1E 6BT, United Kingdom; b Department of Genetics, Evolution and Environment, University College London, London, WC1E 6BT, United Kingdom; c Institute of Molecular Biology and Genetics, National Academy of Sciences of Ukraine, Kyiv, 03680, Ukraine

**Keywords:** Coenzyme A, Protein CoAlation, Oxidative stress, Reactive oxygen species (ROS), *Dictyostelium discoideum*, Morphogenesis

## Abstract

*Dictyostelium discoideum* (*D. discoideum*) is a simple eukaryote with a unique life cycle in which it differentiates from unicellular amoebae into a fruiting body upon starvation. Reactive oxygen species (ROS) have been associated with bacterial predation, as well as regulatory events during *D. discoideum* development and differentiation. Coenzyme A (CoA) is a key metabolic integrator in all living cells. A novel function of CoA in redox regulation, mediated by covalent attachment of CoA to cellular proteins in response to oxidative or metabolic stress, has been recently discovered and termed protein CoAlation. In this study, we report that the level of CoA and protein CoAlation in *D. discoideum* are developmentally regulated, and correlate with the temporal expression pattern of genes implicated in CoA biosynthesis during morphogenesis. Furthermore, treatment of growing *D. discoideum* cells with oxidising agents results in a dose-dependent increase of protein CoAlation. However, much higher concentrations were required when compared to mammalian cells and bacteria. Increased resistance of *D. discoideum* to oxidative stress induced by H_2_O_2_ has previously been attributed to high levels of catalase activity. In support of this notion, we found that H_2_O_2_-induced protein CoAlation is significantly increased in CatA-deficient *D. discoideum* cells. Collectively, this study provides insights into the role of CoA and protein CoAlation in the maintenance of redox homeostasis in amoeba and during *D. discoideum* morphogenesis.

## Highlights

• *D. discoideum* cells are professional phagocytes and produce ROS for efficient bacterial killing.
• *D. discoideum* cells are highly resistant to oxidative stress.
• CoA biosynthetic genes are transcriptionally regulated during morphogenesis.
• The level of CoA and protein CoAlation are developmentally regulated.
• Oxidising agents induce protein CoAlation in *D. discoideum* cells.

## 1 Introduction

Coenzyme A (CoA) is a fundamental and ubiquitous cellular cofactor, and functions as a carbonyl-activating group and an acyl group carrier in diverse biological processes. CoA and its thioester derivatives (acetyl-CoA, malonyl-CoA, succinyl-CoA, HMG-CoA etc.) are involved in a wide range of biochemical reactions, including fatty acid metabolism, protein acylation, biosynthesis of amino acids and cholesterol, and the regulation of gene expression [1,2]. The CoA biosynthetic pathway is conserved across eukaryotes and prokaryotes and involves enzymatic conjugation of pantothenate (vitamin B5), cysteine and adenosine triphosphate (ATP) in five consecutive steps. It is modulated in different ways, including the expression of genes encoding for biosynthetic enzymes, regulation of their enzymatic activities, interconversion among CoA and its thioester derivatives and CoA degradation. Pantothenate kinase (Pank) is the primary and rate-limiting enzyme in CoA biosynthesis and its activity is regulated via feedback inhibition by CoA and its thioester derivatives [1,3]. The enzyme activity can also be modulated by the cell's energy status, as high levels of ATP can displace CoA or its thioesters and support Pank activity [4]. The activity of CoA synthase, which mediates the last two steps of CoA biosynthesis, is also regulated by phospholipids and signalling pathways induced by extracellular stimuli and stresses [[5], [6], [7], [8]]. Dysregulation of CoA biosynthesis and homeostasis has been linked to human pathologies such as cancer, diabetes, neurodegeneration and cardiac hypertrophy [1,3].

Recent studies from our laboratory have uncovered a novel unconventional function of CoA in redox regulation in mammalian and prokaryotic cells, which we termed protein CoAlation [9,10]. It is a reversible and widespread post-translational modification of proteins, involving covalent attachment of CoA via its thiol group to reactive surface exposed thiol groups of cysteine residues in response to oxidative and metabolic stress [11]. To study protein CoAlation we have developed: a) unique anti-CoA monoclonal antibodies; b) a robust procedure for the identification of CoAlated proteins via liquid chromatography tandem mass spectrometry (LC-MS/MS) and c) *in vitro* CoAlation and deCoAlation assays. To date, over one thousand CoAlated proteins have been identified in mammalian cells and tissues, and bacteria under various stress conditions [11].

*D. discoideum* is a soil-dwelling amoeba that has a unique life cycle with motile unicellular and multicellular phases. It is a powerful eukaryotic model organism for biomedical research, and is amenable to investigations of cellular processes, ranging from basic cell biology to growth and differentiation [12]. Single-celled *D. discoideum* amoebae consume bacteria by phagocytosis and proliferate by binary fission. Upon starvation, single vegetative cells cease growth and initiate a programme of multicellular development. This begins with aggregation of cells due to migration towards the secreted chemoattractant cyclic AMP. Aggregation leads to the creation of a tipped mound that further extends to form a finger. The finger forms a slug, which migrates thermotactically and phototactically. Finally, a fruiting body is formed which consists of terminally differentiated dead stalk cells and viable spore cells. The stalk cells are thought to help spore dispersal, when upon the availability of nutrients, they germinate into amoeba thus renewing the life cycle.

Being professional phagocytes, vegetative *D. discoideum* cells (or innate immune cells found in the slug) produce large quantities of toxic ROS for efficient bacterial killing [13,14]. It is thought that extensive exposure to ROS has led *D. discoideum* to evolve high levels of resistance to oxidative stress. Indeed, they express several different catalases and superoxide dismutases during vegetative growth, as well as at specific stages of the developmental cycle. Reactive oxygen species are also thought to play roles during the multicellular stages of development in *D. discoideum*. Manipulation of ROS levels can accelerate or inhibit aggregation [15]. Finally, terminally differentiated spore cells exhibit high levels of resistance to oxidative stress, which is dependent on the expression of a catalase specifically expressed in mature spore cells [16].

*D. discoideum* cells naturally encounter oxidative stress during unicellular growth, multicellular development and terminal spore cell differentiation and dormancy. Understanding the mechanisms underlying their high resistance to oxidative stress remains a key question. It thus represents an ideal system to explore the extent to which protein CoAlation, and its role in oxidative stress responses is conserved in eukaryotes. Here we demonstrate that genes involved in CoA biosynthesis are differentially expressed during growth and morphogenesis, and this pattern correlates with the level of CoA and protein CoAlation. We also found that exposure of *D. discoideum* to oxidative stress resulted in an increase of protein CoAlation. However, much higher concentrations of oxidising agents were required to induce protein CoAlation in *D. discoideum*, when compared to mammalian cells and bacteria [9,10]. Increased resistance to oxidative stress has been attributed to a very high level of catalase activity. Indeed, we found that H_2_O_2_-induced protein CoAlation is significantly increased in CatA-deficient *D. discoideum* cells. Taken together, these findings indicate that changes in the level of CoA and protein CoAlation are associated with redox regulation when *D. discoideum* cells are exposed to oxidative and metabolic stress.

## 2 Materials and methods

### 2.1 CoA antibodies

*Anti*-CoA monoclonal antibodies were previously reported [17]. The following antibodies and dilutions were used in Western blotting: anti-CoA, 1F10 (1:6000 dilution); anti-GSH (rabbit polyclonal, 1:2000 dilution, Abcam); and secondary goat anti-mouse immunoglobulin (IgG) conjugated to AlexaFluor680 (1:10000 dilution, Life Technologies) supplemented with 0.02% sodium dodecyl sulphate (SDS).

### 2.2 Cell culture

*D. discoideum* cells were cultured in suspension in axenic HL5 medium (ForMedium) supplemented with vitamin B12 (0.6 μg/ml) and folate (0.2 μg/ml) at 22 °C. Cells of the Ax3 strain were used in experiments involving treatment with oxidising agents. Morphogenesis studies were performed using Ax4 cells. HEK293 cells and HEK293 cells stably overexpressing Pank1β (HEK293/Pank1β) were maintained in DMEM (Lonza) supplemented with 50 U/ml penicillin, 0.25 μg/ml streptomycin (Lonza) and 10% foetal bovine serum (FBS, Hyclone) in 5% CO_2_ and 37 °C.

### 2.3 Oxidative stress induction

Cells in mid-log phase were collected by centrifugation for 4 min at 2200 rpm and washed three times to remove folate, which can act as antioxidant and neutralise oxidising agents [18]. Cells were re-suspended at a cell density of 2.5 × 10^6^ cells/ml in vitamin-free HL5 media and shaken at 180 rpm at 22 °C for 60 min. This was followed by treatment for 30 min with diamide, H_2_O_2_ or t-butylhydroperoxide (TBH). Cells were collected via centrifugation at 3500 rpm and 4 °C and frozen on dry ice.

### 2.4 Cell development

Log phase *D. discoideum* cells were collected by centrifugation and washed with KK2 potassium phosphate buffer (20 mM K_2_HPO_4_, KH_2_PO_4_, pH6.8) before re-suspending at a density of 1 × 10^8^ cells/ml in KK2. 3 × 10^7^ cells were evenly spread over the surface of a freshly prepared 10 cm agar plate (20 ml of 1.5% agar in KK2) to induce starvation and initiate differentiation. Plates were incubated in a humid box at 22 °C in the dark before harvesting by centrifugation. Cells were instantly frozen in dry ice.

### 2.5 Cell lysis and Western blot analysis

Cell pellets were re-suspended in ice-cold lysis buffer (containing 50 mM Tris-HCl pH 7.5, 150 mM NaCl, 5 mM tetra-sodium pyrophosphate, 50 mM sodium fluoride, 5 mM ethylenediaminetetraacetic acid (EDTA), and 1% Triton X-100) supplemented with freshly prepared 100 mM *N*-ethylmaleimide (NEM) and 2 x Protease Inhibitor Cocktail (PIC, Roche). To lyse spores, 0.1 mm diameter silica-zirconia beads (Biospec Products) and 2 mm Tungsten Carbide beads (Qiagen) were added and placed in a Tissue Lyser II (Qiagen) set at 30 Hz for 2 min. Total cell lysates were centrifuged for 20 min at 14000 rpm and 4 °C to remove the insoluble fraction. Protein concentration of the supernatant was measured by Bicinchoninic acid (BCA) Protein Assay Kit (Pierce ThermoFisher Scientific). Samples of cell lysates (∼40 μg of protein) were separated by SDS-PAGE on 10% TruPAGE Precast Gels and immunoblotted with anti-CoA mAb as previously described [9].

### 2.6 Analysis of CoA levels

#### 2.6.1 Extraction of CoA and the recycling assay

The method to extract CoA from *Bacillus megaterium* was used in this study [19]. CoA extracts were re-suspended in 1 mM DTT and centrifuged for 5 min at 14 000 rpm and 20 °C. CoA level was measured by a modification of the recycling assay developed by Allred and Guy [20,21]. Sets of CoA standards (0.03 μM, 0.1 μM, 0.3 μM, 1 μM, 3 μM, 9 μM) were freshly prepared by serial dilutions of a standard stock solution in 10 mM potassium phosphate buffer (pH 7) and 1 mM DTT.

#### 2.6.2 Normalisation of extracts through measurement of protein concentration

The level of CoA in cell cultures is commonly standardised per total protein [22]. To determine the total amount of protein in the sample from which CoA was extracted, the sample in extraction buffer was mixed with 6 M guanidine chloride and vortexed until the solution became transparent. The fruiting body sample was lysed in a Tissue Lyser II (Qiagen) set at 30 Hz for 2 min, using 0.1 mm diameter silica-zirconia beads (Biospec Products) and 2 mm Tungsten Carbide bead (Qiagen). The samples were centrifuged for 5 min at 14 000 rpm at RT and the BCA assay was used to measure protein concentration.

### 2.7 Statistical analysis

The concentration of CoA in the samples was extrapolated from the calibration curve and calculated as pmol/mg of total protein (mean ± standard error of the mean (SEM)). Paired *t*-test was used to compare level of CoA between developmental stages. N represents the number of independent experiments. Significant values were considered at p-value <0.05.

## 3 Results

### 3.1 Transcriptional regulation of CoA biosynthetic genes

**Fig. 1:**
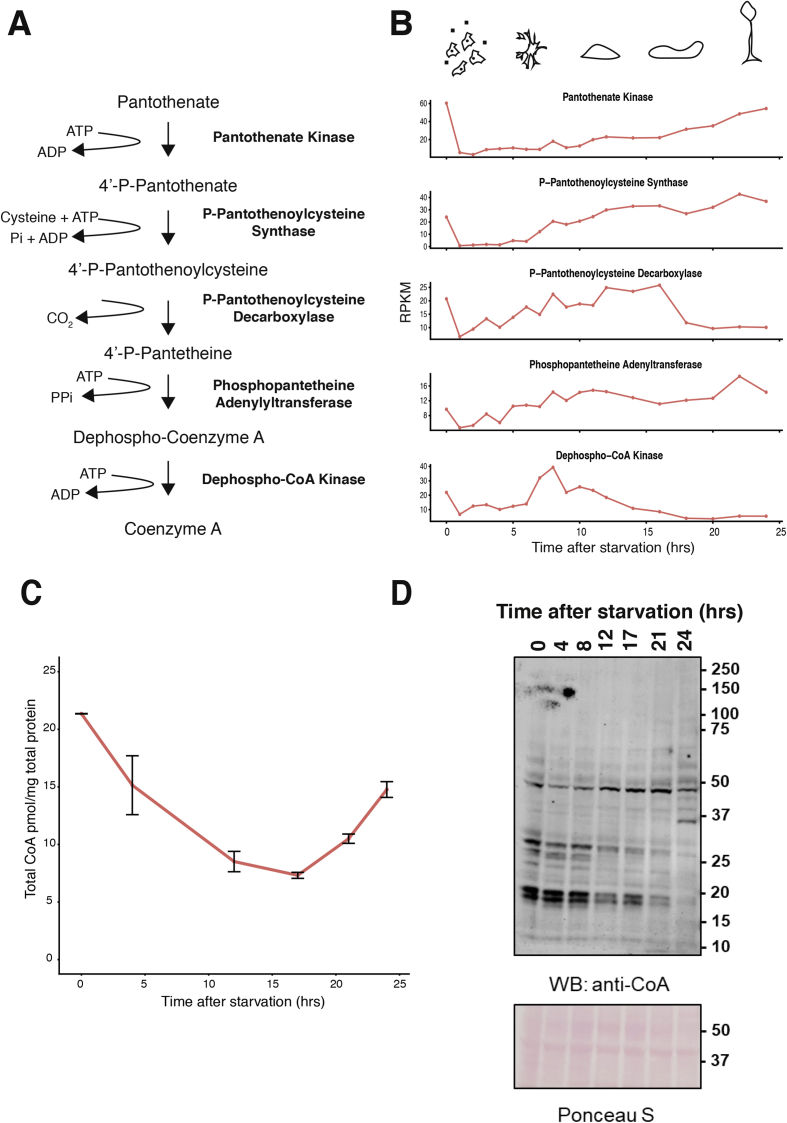
The expression of genes involved in CoA biosynthesis, the level of CoA and protein CoAlation in *D. discoideum* are developmentally regulated. **A.** Schematic depicting the evolutionary conserved CoA biosynthetic pathway. **B.** Transcriptional profiling of genes involved in the CoA biosynthetic pathway during growth and development. **C.** Analysis of total CoA level at developmental stages by an enzymatic recycling assay. Data represent mean ± SEM. *p < 0.05 compared to mound, slug culminant and fruiting body stages, ≠ p < 0.05 compared to slug and culminant stages. **D.** Western blot analysis of protein CoAlation during vegetative growth and morphogenesis. Protein CoAlation was examined by immunoblotting with anti-CoA antibody.

Protein CoAlation has been suggested to play a role in oxidative stress responses. *D. discoideum* cells are likely to be exposed to oxidative stress at different stages of their life cycle. We therefore tested whether protein CoAlation may play a role in this system. Firstly, we examined the expression profile of genes required to produce CoA in *D. discoideum* at different stages of its life cycle (Fig. 1A). We found that all genes were developmentally regulated, with high expression levels in vegetative cells (Fig. 1B). Levels then fall upon starvation, but rise again during multicellular development. This pattern is most evident for Pank, which encodes the rate-limiting enzyme in the CoA biosynthetic pathway. Pank expression was highest in growing cells, then dropped significantly upon starvation, but gradually increased again during the developmental stages (Fig. 1B), reaching a peak during culmination that was comparable to that seen in vegetative cells. These findings support the idea that changes in cellular metabolism and redox regulation could modulate the production of CoA during morphogenesis.

### 3.2 Levels of CoA in *D. discoideum* cells

The expression pattern of genes required for CoA biosynthesis suggested that CoA levels may in turn be developmentally regulated. To test this hypothesis, we determined whether the total level of CoA in *D. discoideum* cells correlated with Pank mRNA levels. The extraction of CoA from *D. discoideum* was found to be efficient and experiments with internal standards showed that the recycling assay can measure CoA levels reliably in the picomolar range (Suppl. Figure 1A). The concentration of CoA in vegetative *D. discoideum* (45 pmol/mg total protein) cells was found to be comparable to that in HEK293 cells (18 pmol/mg total protein) and, as anticipated, less than in HEK293/Pank1β cells (381 pmol/mg total protein) (Suppl. Figure 1B). We next extracted and measured CoA from *D. discoideum* cells at different developmental stages. CoA levels were found to follow levels of Pank mRNA expression, decreasing after starvation but then rising again during multicellular development (Fig. 1C). CoA levels in the fruiting body stage (24 h of starvation) were statistically significantly higher than the CoA levels of slug, and culminant stages (17 h, and 21 h of starvation, respectively), which correlated with Pank mRNA expression profile.

### 3.3 Analysis of protein CoAlation during morphogenesis

Recent studies from our laboratory have revealed that the extent of protein CoAlation induced by oxidising agents and metabolic stress is determined by the level of CoA in cells or tissues [9]. Because there are significant fluctuations in CoA levels across different stages of morphogenesis, we immunoblotted these corresponding protein samples with anti-CoA antibody under non-reducing conditions. A distinctive pattern of CoA-modified proteins was observed at different developmental stages (Fig. 1D). Significant protein CoAlation was detected in vegetative cells, especially for a number of proteins in the lower molecular weight region. The signal intensity of these proteins around 50 kDa dropped after the initiation of starvation (aggregation stage), but then gradually increased to reach the maximum at the fruiting body stage. However, for other proteins, CoAlation increased during multicellular development, with a unique pattern evident upon fruiting body formation and spore cell differentiation (Fig. 1D**)**.

### 3.4 Oxidising agents induce protein CoAlation in *D. discoideum* cells

**Fig. 2:**
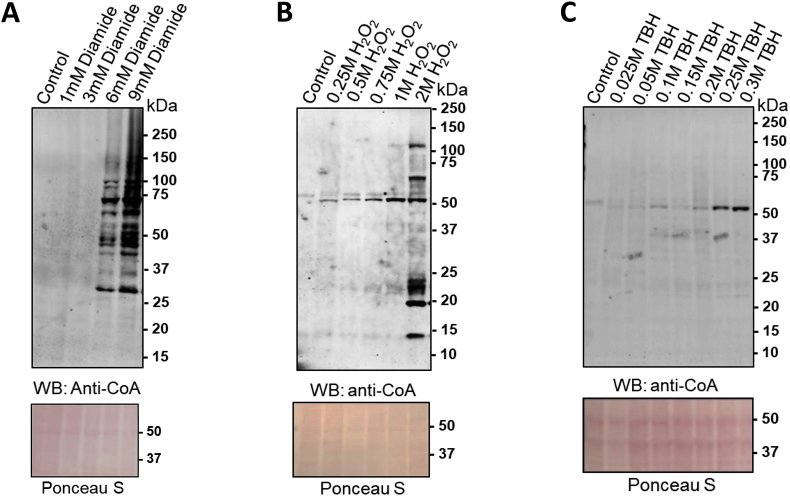
Dose-dependent induction of protein CoAlation in *D. discoideum* cells in response to diamide (**A, B**), H_2_O_2_ (**C, D**) and TBH (**E, F**). Exponentially growing cells were treated or not treated with diamide, H_2_O_2_ and TBH at the indicated concentrations for 30 min. Cells were lysed and protein CoAlation was examined by immunoblotting with anti-CoA antibody.

The expression of the Pank gene, the level of CoA and protein CoAlation all change during *D. discoideum* morphogenesis. Previous studies have revealed that protein CoAlation is induced in mammalian and bacterial cells exposed to oxidising agents [9,10]. We therefore investigated whether covalent protein modification by CoA is also associated with oxidative stress responses in *D. discoideum*. Taking into account that *D. discoideum* cells exhibit high resistance to oxidative stress [23], we examined protein CoAlation in a dose-dependent study using relatively high concentrations of oxidising agents. Growing cells were treated with H_2_O_2_, diamide or TBH. Cell lysates were prepared and protein extracts separated by SDS-PAGE. Western blot analysis with anti-CoA antibody revealed an increase in protein CoAlation with all treatments compared to control cells (Fig. 2 and Suppl. Figure 2). Levels of protein CoAlation were extensive when cells were treated with diamide, but much weaker with H_2_O_2_ and TBH even at very high doses (compared to those used in mammalian cells to induce oxidative stress). Immunoreactive bands were detected in control cells and cells exposed to 1 mM and 3 mM diamide, and protein CoAlation increased markedly when cells were treated with 6 mM or 9 mM diamide (Fig. 2A). In contrast, H_2_O_2_- and TBH-induced oxidative stress caused very weak protein CoAlation with up to 1M H_2_O_2_ and 0.3 M TBH in the culture medium (Fig. 2B and C). The above results suggest that *D. discoideum* cells employ different antioxidant defence mechanisms to cope with oxidative stress induced by diamide and H_2_O_2_.

### 3.5 Protein CoAlation is strongly induced in CatA-deficient cells treated with H_2_O_2_

**Fig. 3:**
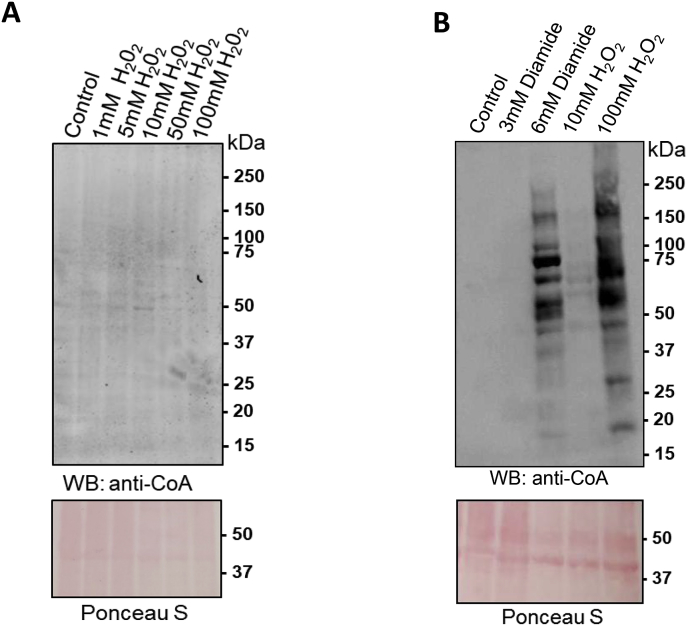
Analysis of protein CoAlation in wild type and CatA deficient cells in response to TBH. Exponentially growing Ax3 (**A**) and CatA deficient (**B**) cells were treated or not treated with H_2_O_2_ at the indicated concentrations for 30 min. Cells were lysed and protein CoAlation was examined by immunoblotting with anti-CoA antibody.

To deal with redox active species, *D. discoideum* cells produce antioxidant enzymes, such as superoxide dismutase, catalase, and glutathione peroxidase. *D. discoideum* expresses two catalase genes (catA and catB), which are differentially regulated both temporally and spatially during morphogenesis [16]. The catA gene is expressed throughout growth and development, while the catB gene is only expressed at the fruiting body stage and spore formation. *D. discoideum* mutant cells with significantly reduced levels of catA mRNA and enzyme have been shown to exhibit markedly increased sensitivity to H_2_O_2_ [24]. We therefore compared the level of protein CoAlation in wild type and CatA deficient *D. discoideum* cells. As shown in Fig. 3A and B, protein CoAlation was markedly increased in CatA deficient cells treated with low doses of H_2_O_2_. These results indicate that CatA efficiently neutralises exogenous and endogenous H_2_O_2_, and protects *D. discoideum* cells from oxidative damage. Thus, in contrast to diamide, the involvement of low molecular weight (LMW) thiols, such as CoA, in antioxidant defence induced by H_2_O_2_ may normally be minimal.

## 4 Discussion

*D. discoideum* cells alternate between growing as single cells and developing as a multicellular organism after chemotactic aggregation induced by starvation. The expression of several thousand genes governs the programme of morphogenesis [25,26]. In this study we report the transcriptional regulation of genes involved in CoA biosynthesis during the life cycle of *D. discoideum*. We found that all genes in the CoA biosynthetic pathway are developmentally regulated, showing high expression levels in vegetative cells, then falling dramatically upon starvation, before gradually increasing again during multicellular development. This pattern is most apparent for Pank. Studies in mammalian cells have revealed Pank is a rate-limiting enzyme in the CoA biosynthetic pathway, as overexpression of Pank1β alone is sufficient to significantly increase CoA production in Cos7 and HEK293 cells [9,27]. Similarly, measurements of total level of CoA during normal growth and development in *D. discoideum* revealed a close correlation with the level of transcripts for CoA biosynthetic genes, especially Pank.

Why is the level of CoA in *D. discoideum* developmentally regulated? During vegetative growth, *D. discoideum* cells require large quantities of CoA to produce a diverse range of thioester derivatives (acetyl CoA, malonyl CoA etc.) which play a central role in catabolic and anabolic processes. The findings of this study suggest that CoA, in addition to its well-established functions in cellular metabolism, is also involved in redox regulation in *D. discoideum*. One reason for this may be that *D. discoideum* cells act as professional phagocytes that use ROS to kill bacteria and obtain nutrients for growth. Indeed, high levels of ROS production have been observed in vegetative cells upon LPS stimulation [13]. Furthermore, the extent of protein CoAlation changes during growth and morphogenesis, and this pattern correlates with phases of the life cycle of *D. discoideum* where redox regulation is likely to be necessary for innate immune cell function or the regulation of morphogenesis [14]. Indeed, ROS production is required for innate immune cell function, whilst low concentrations of ROS have been implicated in regulatory signalling pathways and numerous physiological processes during morphogenesis of a variety of systems [15]. Exposure to oxidative stress is thus likely to be a normal feature of the *D. discoideum* life cycle, and the ability of these cells to withstand oxidative stress by employing different antioxidant defence strategies, including LMW thiols, is likely crucial for their success.

We propose that covalent protein modification by CoA during the life cycle of *D. discoideum* has the potential to become an integral part of redox sensing and regulation by: a) modulating the activity, stability and subcellular localisation of modified proteins; b) generating a unique binding motif for intra- and inter-molecular interactions; and c) promoting the formation of regulatory signalling complexes. Finally, protein CoAlation may also contribute to maintaining metabolic dormancy of terminally differentiated spore cells which are highly resistant to diverse environmental stress conditions, including oxidative damage.
