# Supplementary figures and images for: Coenzyme A and protein CoAlation levels are regulated in response to oxidative stress and during morphogenesis in Dictyostelium discoideum
Source: Biochem Biophys Res Commun. 2019 Apr 2;511(2):294–9. doi: 10.1016/j.bbrc.2019.02.031 (PMC6416166; doi:10.1016/j.bbrc.2019.02.031)

**A**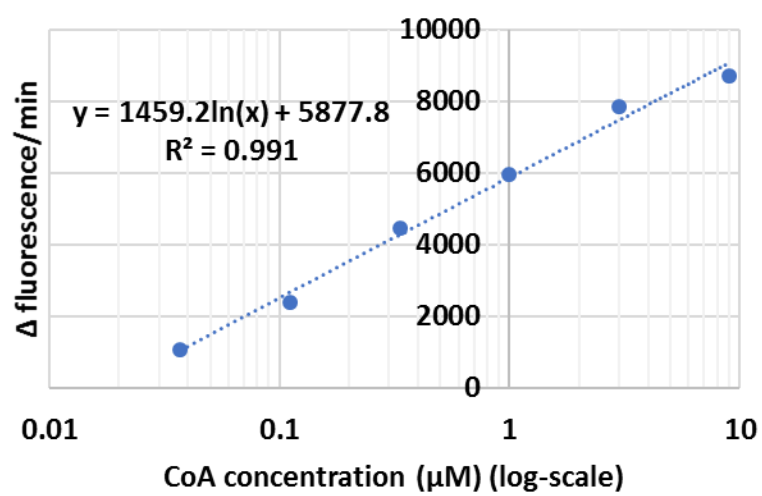**B**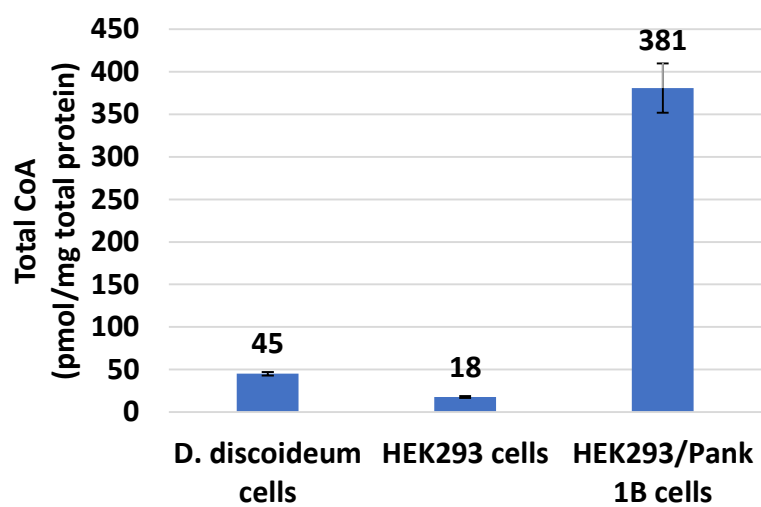

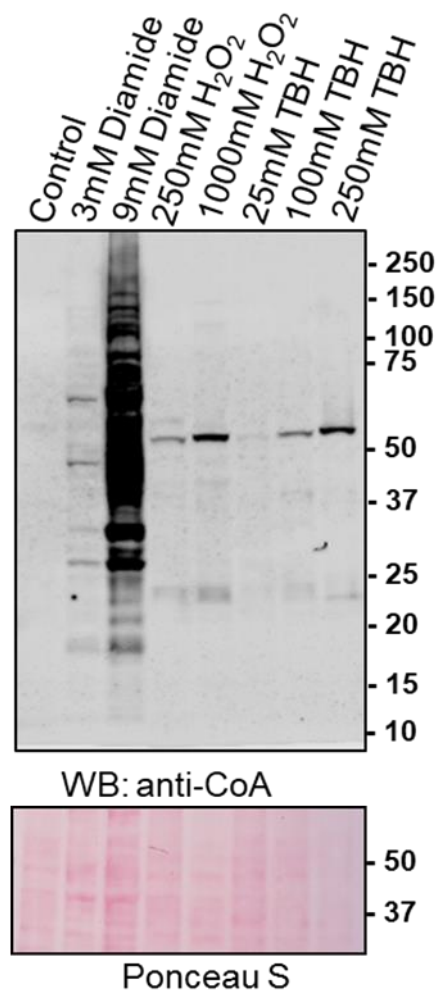

Supplement: Multimedia component 2 [file mmc2.pdf]
